# Dietary Bioactive Compounds: Implications for Oxidative Stress and Inflammation

**DOI:** 10.3390/nu15234966

**Published:** 2023-11-30

**Authors:** Doretta Cuffaro, Maria Digiacomo, Marco Macchia

**Affiliations:** 1 Department of Pharmacy, University of Pisa, 56126 Pisa, Italy; doretta.cuffaro@unipi.it (D.C.); marco.macchia@unipi.it (M.M.); 2 Interdepartmental Research Center “Nutraceuticals and Food for Health”, University of Pisa, 56100 Pisa, Italy

Nowadays, it has been amply demonstrated how an appropriate diet and lifestyle are essential for preserving wellbeing and preventing illnesses [1,2]. Dietary bioactive substances are natural constituents present in food that provide health benefits. These compounds can be defined as substances capable of modulating biological activities and important physiological functions [3,4,5]. It is known that the consumption of dietary bioactive compounds, especially phytochemicals, contribute to decreasing the risk of chronic disorders, such as cancer and cardiovascular and neurodegenerative diseases [6,7,8,9,10].

In the last few years, the study of the efficacy of these natural bioactive compounds has attracted the increasing interest of researchers. In this regard, this Special Issue in *Nutrients* entitled “Dietary Bioactive Compounds: Implications for Oxidative Stress and Inflammation”, which includes a review and 12 original articles, provides an insight on current knowledge about the nutraceutical effects of different bioactive compounds present in foods or their related byproducts on some pathologies, with a special focus on their antioxidant and anti-inflammatory properties.

In particular, the role of various bioactive-rich extracts on gastrointestinal disorders [11] have been further discussed, as documented by three papers that deal with the effects of a number of natural extracts in pathologies affecting the digestive system.

Lo et al. [12] studied the potential treatment of ulcerative colitis (UC) with the oral administration of ethanol extracts from rice bran and whole-grain adlay seeds, in mice with dinitrobenzene sulfonic acid (DNBS)-induced UC. Their results indicated that rice bran ethanol extract reduces UC-induced damage in the colon along with inflammation and oxidative stress in DNBS-induced UC mice. Moreover, whole-grain adlay seed ethanol extract is able to modulate colonic inflammation and clinical symptoms in UC mice. Additionally, both of the extracts reversed DNBS-induced alterations in T-helper-cell-associated cytokines and glutathione in the colon. Unfortunately, in this paper, the composition of the ethanol extracts was not defined; thus, the main bioactive compound(s) in rice bran and whole-grain adlay seeds responsible for their activities was not identified.

Chen et al. [13] explored the effect of Shibi tea (EST) on liver injury in an in vivo mouse study. EST is a non-Camellia tea prepared by the infusion of dried *Adinandra nitida* leaves, which are rich in flavonoids and especially in Camellianin A (CA) [14]. In this work, for the first time, the benefit of EST and CA in liver injury was investigated, with the authors exploiting the hepatoprotective effects of EST and CA extracts in a carbon tetrachloride (CCl_4_)-induced acute-liver-injury mouse model. Additionally, the anti-inflammatory, anti-apoptosis, and antioxidative effects of EST and CA in repairing acute liver injury were explored, with the authors analyzing the regulation of the oxidative stress signaling pathways and the expression of inflammatory cytokines and phosphorylated nuclear factors. The results highlight that EST and CA display anti-inflammatory, anti-apoptosis, and antioxidative properties, and could be promising agents in the prevention of liver injuries.

Liu et al. [15] evaluated the role of C-phycocyanin (CPC) and *Lycium barbarum* polysaccharides (LBP) on aspirin-induced gastric damage in rat gastric mucosal (RGM-1) cells. The primary active compounds, CPC and LBP, found in Spirulina platensis and wolfberry, respectively, possess antioxidative, anti-inflammatory, and immunoregulatory properties [15,16]. Therefore, the aim of this study was to use these food ingredients to contrast the gastric damage caused by the (i.e., acetyl salicylic acid) chronic administration of aspirin, for anti-inflammatory or cardiovascular purposes [17]. The evaluation of CPC and LBP at a high dose of 500 μg/mL demonstrated a promising ability to attenuate aspirin-gastric injury in gastric RGM-1 cells. CPC and LBP affected the activation of ERK and JNK signaling pathways, increasing the expression of the anti-inflammatory interleukin 10 (IL-10) and modulating proinflammatory markers (NF-κB, caspase 3, Bax protein). In particular, CPC and/or LBP exhibited anti-inflammatory effects by inhibiting activation of the ERK signaling pathway, while LBP reduced apoptosis by decreasing activation of the JNK signaling pathway in gastric RGM-1 cells with aspirin-induced epithelial damage.

In other diseases, such as skin disorders, the health benefits of bioactive compounds contained in natural extracts were exploited [18,19,20,21]. In fact, many studies discussed the role of natural extracts in skin disorders and in particular in atopic dermatitis (AD) [22,23,24]. In this Special Issue, Bae et al. [25] exploited the effect of *Daphnopsis costaricensis* extract on an in vivo AD lesion model. This paper outlines the impact of the *D. costaricensis* EtOH extract (DCE) on AD-like lesions in a mouse model induced with oxazolone (OX). The findings indicate that DCE significantly improved AD-like pathology by reducing ear epidermal thickness and mast cell infiltration. Additionally, eleven compounds, with a flavonoid-like structure, were isolated and identified in extract, through the use of 1D and 2D NMR and HR-MS data. These compounds have been evaluated for their anti-inflammatory and anti-allergic activities, demonstrating that 7,8-dimethoxyflavone and 7,2′-dimethoxyflavone were able to inhibit IL-4 overproduction and mast cell degranulation in vitro (in a rat basophilic leukemia cell line, RBL-2H3 cells). Therefore, DCE could be useful in the treatment of AD.

Beyond the nutraceutical properties widely demonstrated of a lot of foods, recently the study of agri-food wastes, as a source of bioactive compounds, has also received growing interest [26,27,28,29]. Moreover, these wastes are related to an authentic environmental drawback, and consequently waste disposal becomes a challenge in the agri-food industrial economy [30,31,32,33]. Different residues, such as pomace, leaves, wastewaters, peels, stems or flowers are discarded and a real effort is being placed on the valorization of food chain byproducts [34]. The re-use of agrifood wastes as an a renewable, abundant and low-cost source for the production of high value products, is presently being exploited. In this Special Issue, seven papers explored the important valorization of food byproducts, giving a wide point of views on this in-trend topic.

Two papers investigated the valorization of two main byproducts of extra virgin olive oil (EVOO) production: olive mill wastewater (OMWW) and olive leaves. The nutraceutical proprieties of EVOO are already well known and confer a leading role to EVOO in the health benefits of the Mediterranean diet [35]. Despite dietary benefits, EVOO production has a great impact in terms of sustainability due to the difficult management of its related wastes such as olive leaves and OMWW [36,37,38,39,40]. These discards contain phenolic compounds endowed with nutraceutical properties and they are a promising font of bioactive compounds. Therefore, correct waste management, which considers all potential paths for a circular economy of the olive oil supply chain, is crucial.

Cuffaro et al. [41] reported the study of improved nutraceutical properties of EVOO extract by adding different percentages of olive leaf extracts (OLE). In this study, a promising EVOO extract enriched by 8% OLE was selected. This extract contained important nutraceutical polyphenols such as oleocanthal, oleacein and oleuropein in similar quantities, as reported by HPLC analysis. The 8% OLE-EVOO extract showed increased antioxidant and antiradical properties (evaluated by in vitro assays (DPPH, ABTS and FRAP)) with respect to the EVOO extract, evidencing a potential effect in reducing oxidative stress. Moreover, its anti-inflammatory activity was evaluated in terms of cyclooxygenase (COX) enzyme inhibition. The 8% OLE-EVOO extract displayed four-fold improved COX-1 inhibition and two-fold COX-2 inhibition (COX-1 IC_50_ = 475 μg/mL; COX-2 IC_50_ = 383 μg/mL) with respect to the EVOO extract (COX-1 IC_50_ = 1.90 mg/mL; COX-2 IC_50_ = 900 μg/mL).

The same research group studied the potential beneficial effect of bioactive polyphenols in OMWW extracts [42]. The aim of this study was to point out a possible nutraceutical valorization of this byproduct, exploiting its biological properties. Similarly to EVOO [43], the composition of OMWW varied depending on the olive cultivar and extraction system. This study assessed multiple samples of three-phase extraction OMWWs obtained from two cultivars of olives, Leccino (CL) and Frantoio (CF), collected in October (CF1 and CL1) and November (CF2 and CL2). The polyphenolic profile (18 polyphenols) of OMWW extracts was defined using quali-quantitative analysis performed by LC-MS/MS, revealing high amount of tyrosol and hydroxytyrosol in all the samples, and oleacein in the October samples, rarely found in OMWW. Furthermore, the antioxidant profile and the anti-inflammatory effect in terms of COX 1 and COX2 inhibition were evaluated, resulting in significantly low values of IC_50_ COX-2 inhibition for oleacein-rich extracts (ranging 0.080 mg/mL).

Similarly to EVOO, the beer industry is environmentally affected by the difficult management of byproducts [44,45,46,47], especially brewers’ spent grain (BSG). BSG is the most abundant byproduct of the brewing industry, composed mainly of dietary fiber (50%), proteins (30%), and bioactive compounds, such as hydroxycinnamic acids [48,49,50]. In this Special Issue, Gutierrez-Barrutia [51] investigated how extrusion process, a thermomechanical procedure characterized by high temperature and pressure for a short period of time, influences the bioaccessibility of BSG nutrients (glucose and amino acids) and non-nutrients (phenolic compounds). The study revealed that the extrusion process did not affect glucose bioaccessibility or gluten digestibility, favoring amino acid release during digestion. On the other hand, significantly improved gastrointestinal and colonic bioaccessibility of BSG phenolic compounds was achieved in extruded BSG. Moreover, extruded BSG intestinal digests inhibited glucose transport through increased α-glucosidase and α-amylase inhibition, compared to untreated BSG. Additionally, extruded BSG intestinal digests inhibited intracellular ROS formation and showed anti-inflammatory properties, unlike untreated BSG. Therefore, the extrusion process improved the nutritional value and biological properties of BSG, so the extruded BSG has the potential to be a sustainable and promising ingredient with positive properties that could be useful in both the prevention and treatment of various non-communicable diseases.

The valorization of byproducts affects not only food production, but also fruit collection [52,53] such as cherry, tangerine and chestnut, and edible mushrooms [54,55]. This Special Issue explored all these different fields of applications thanks to the various reported studies.

Nunes et al. [56] discussed the anti-inflammatory and antimicrobial potential of different extracts from the stems, leaves and flowers of *Prunus avium* L., a cultivar of Portuguese cherry from the Fundão region [57,58]. The hydroethanolic extracts of leaves and stems and the aqueous infusion of flowers were effective in reducing inflammation in terms of a decreased level of NO production on a lypopolisacharide (LPS)-stimulated mouse macrophage (RAW 264.7) cell line. Furthermore, the aqueous infusions of all by-products, especially cherry flowers, demonstrated scavenging activity against NO radicals. Moreover, the leaf extracts showed an antimicrobial effect against most of the tested bacteria.

Tangerine peel (*Citrus reticulatae Pericarpium*, CRP) is the main byproduct in the cultivation of tangerine [59,60,61]. Dried tangerine peel is a traditional Chinese medicine already studied for its nutraceutical properties. Its main components are volatile oils and flavonoids such as hesperidin, naringenin, nobiletin, and tangeretin [61]. Here, Wang et al. [62] discussed the protective effect of Tangerine peel extract in endothelial dysfunction and vascular inflammation related to diabetes on AMPK activation in a rat model. CRP extract was orally administered for 4 weeks at 400 mg/kg/day in high-fat diet/streptozotocin (HFD/STZ)-induced diabetic rats. After treatment, the rats successfully reversed all the diabetic symptoms, resulting in a normalized blood pressure and plasma lipid profile, as well as plasma levels of liver enzymes in diabetic rats. The CRP extract also suppressed vascular inflammatory markers, inducing AMPK activation in the aortas of diabetic rats. Therefore, administering tangerine peel extract chronically can safeguard arteries from vascular inflammation and endothelial dysfunction in diabetic rats.

*Flammulina velutipes* (FV) is an edible mushroom presenting nutritional and medicinal values. FV mycorrhizae is a poorly studied by-product of FV representing an important source of bioactive compounds [63,64]. In their study, Luo et al. [65] investigated, at first, the composition of FV mycorrhizae, and secondly its effects on lipid metabolism and inflammation in perirenal adipose tissue (PAT), which is still unknown. The composition of FV mycorrhizae comprehend multifarious nutritive components among them polysaccharide, amino acids and derivatives, and organic compounds. HFD supplemented with 4% FV mycorrhizae (HFDFV) caused the attenuation of HFD-induced lipid disorders, reducing HFD-induced oxidative stress and pro inflammatory cytokines, both in the liver and perirenal adipose tissue (PAT) of mice. These results indicated the promising application of FV mycorrhizae as a functional food and herbal medicine in the treatment of obesity. 

Piazza et al. [66] described, for the first time, the nutraceutical potential of *Castanea sativa* Mill. leaf extracts in gastritis caused by *Helicobacter pylori* (*H. pylori*) infection. The authors evaluated the polyphenolic profile in *Castanea sativa* Mill. leaf extracts, with a particular focus on ellagitannins [67,68], a nutraceutical polyphenol class with potential gastroprotective properties. Castalagin and vescalagin were identified and quantified in hydroalcoholic leaf extracts using LC–MS. Thus, the anti-inflammatory and antibacterial activity of leaf extracts, in comparison with pure castalagin, were investigated in a model of human gastric epithelium (GES-1) infected by *H. pylori*. The leaf extract and pure ellagitannins inhibited IL-8 release (IC_50_ ≈ 28 µg/mL and 11 µM, respectively), partially attenuating NF-κB signaling and reducing bacterial growth and cell adhesion. These results were also confirmed by transcriptional studies in which castalagin was able to decrease genes involved in inflammatory pathways (NF-κB and AP-1) and cell migration (Rho GTPase). These observations suggested that *Castanea sativa* Mill. leaves could be adopted to produce sustainable and bioactive extracts.

As seen in the various studies reported in this Special Issue and also in the numerous papers present in the literature, bioactive compounds include a plethora of molecules, such as phenolics, carotenoids, vitamins, minerals, and fibers, ubiquitously expressed in fruits, vegetables, grains and legumes [53]. Usually, the most representative polyphenols in food are phenolic acids, flavonoids and anthocyanins [69,70]. Anthocyanins are a class of flavonoids offering various health benefits, and are responsible for the blue, purple, and red pigments detected in different type of fruits and vegetables [71,72]. These polyphenols exert a wide range of nutraceutical activities, including antioxidant and anti-inflammatory properties, associated with cardioprotection, anti-carcinogenicity or neuroprotection [71]. Among the more than 1000 types of anthocyanins present in the literature, Malvidin is one of the most studied. In this Special Issue, Merecz-Sadowska et al. [73] collected and reviewed the reported studies in the literature investigating the role of malvidin and its related glucosides in different cell, animal and human models. Besides their colorant capacity, malvidin and its related glycosides revealed a widespread range of beneficial properties promoted by antioxidant and anti-inflammatory mechanisms. In addition, these molecules showed the ability to counteract the onset and progression of several diseases whose pathogeneses are linked to oxidative stress. These findings suggest a potential future application for malvidin and its glycosides as ingredients of functional food, able to offer both aesthetic and nutritional advantages.

Despite the extensive studies demonstrating the health effects of bioactive compounds, such as polyphenols, in humans, one of the major drawbacks in the development of single polyphenols or polyphenol-rich natural extracts, as functional ingredients or dietary supplements, is related to their pharmacokinetic profile, compromised by their poor aqueous solubility, intensive metabolism, and low systemic absorption [74,75,76]. Only a few studies have considered the aspects of bioavailability and metabolism [77,78,79]. However, most of these have reported in vitro models of pure compounds in which the matrix effects were not considered [69,80,81].

In this context, innovative strategies could be developed to deliver pure polyphenols or natural extracts rich in bioactive compounds [82,83]. One of the alternative delivery methods, able to increase the solubility of active substances by bypassing metabolism, is represented by electrospun nanofibres incorporating bioactive substances [84,85,86]. In this Special Issue, Paczkowska-Walendowska et al. [87] reported the development of *P. cuspidati* radix extract nanofibers as an innovative approach in solid dispersion for the buccal delivery system. At first, the authors optimized the extraction process in order to obtain the best extract in terms of richness in bioactive compounds, especially stilbenes such as resveratrol and polydantins. The selected *P. cuspidati* radix extract was incorporated in nanofibers based on polyvinylpyrrolidone/cyclodextrin (PVP/HPβCD) using an electrospinning technique, affording nanofibers the six-fold improved solubility of resveratrol and polydantins compared to pure standards. Thus, the electrospun nanofibers may be easily applied within the oral cavity, immediately releasing the incorporated bioactives. These results, along with the intrinsic antioxidant and anti-inflammatory properties of *P. cuspidati* extract, indicate that the buccal delivery system might be an alternative strategy to improve the bioavailability of bioactives.

In conclusion, the set of studies collected in the Special Issue “Dietary Bioactive Compounds: Implications for Oxidative Stress and Inflammation” have explored different aspects of dietary bioactive compounds. The application of different nutraceutical extracts rich in bioactive compounds on gastrointestinal injury and skin disorder have been analyzed. Furthermore, the actual trend topic of agrifood wastes has been widely explored here, with studies analyzing the potentiality of nutraceutical extracts of different byproducts of the food industry (EVOO and beer), fruits (sweet cherry, chestnuts and tangerine) and mushrooms. Moreover, an insight on anthocyanins, an important class of bioactive compound, was proposed. Additionally, aspects related to metabolism and the bioavailability of bioactive compounds have been attended to in proposition of the use of nanofibers.

All these papers highlight that dietary bioactive compounds endowed with antioxidant and anti-inflammatory properties could be beneficial for health and should be further studied to develop functional foods or food supplements.
